# Cyclophilins and nucleoporins are required for infection mediated by capsids from circulating HIV-2 primary isolates

**DOI:** 10.1038/srep45214

**Published:** 2017-03-27

**Authors:** João I. Mamede, Florence Damond, Ariel de Bernardo, Sophie Matheron, Diane Descamps, Jean-Luc Battini, Marc Sitbon, Valérie Courgnaud

**Affiliations:** 1 Institut de Génétique Moléculaire de Montpellier, UMR 5535 CNRS, 1919 route de Mende, 34293 Montpellier cedex 5, France; Université de Montpellier, 163 rue Auguste Broussonnet, 34090 Montpellier, France; 2 Laboratoire de Virologie, AP-HP Groupe Hospitalier Bichat-Claude Bernard, HUPNVS, Université Paris Diderot, Sorbonne Paris Cité, EA4409, 75018, Paris, France

## Abstract

HIV-2 groups have emerged from sooty mangabey SIV and entered the human population in Africa on several separate occasions. Compared to world pandemic HIV-1 that arose from the chimpanzee SIVcpz virus, the SIVsm-derived HIV-2, largely confined to West Africa, is less replicative, less transmissible and less pathogenic. Here, we evaluated the interactions between host cellular factors, which control HIV-1 infection and target the capsid, and HIV-2 capsids obtained from primary isolates from patients with different disease progression status. We showed that, like HIV-1, all HIV-2 CA we tested exhibited a dependence on cyclophilin A. However, we observed no correlation between HIV-2 viremia and susceptibility to hu-TRIM5alpha or dependence to CypA. Finally, we found that all CA from HIV-2 primary isolates exploit Nup358 and Nup153 for nucleus transposition. Altogether, these findings indicate that the ability to use the two latter nucleoporins is essential to infection of human cells for both HIV-1 and HIV-2. This dependence provides another molecular target that could be used for antiviral strategies against both HIV-1 and 2, based on both nucleoporins.

Cross-species transmission of SIV from sooty mangabeys to humans in West Africa gave rise to at least nine HIV-2 groups. HIV-2 remained largely restricted to West Africa^1^ in contrast to the HIV-1 group M that is responsible for the worldwide AIDS pandemic. HIV-1 group M appeared during a zoonosis from chimpanzee. Among the nine known HIV-2 groups, only groups A and B have spread throughout West Africa^1^,^2^,^3^. Globally this suggests a weaker adaptability of HIV-2 in humans. Indeed, in several West African countries, HIV-2 prevalence has been declining while HIV-1 infection has increased^4^,^5^. Despite the fact that HIV-1 and HIV-2 have similar routes of transmissions, cellular targets, or clinical consequences, the characteristics of disease progression in infected patients differ drastically in many ways. The natural course of HIV infection is usually described in three stages (acute, latent and AIDS). However, the incidence of immunodeficiency and its progression are significantly reduced in HIV-2 patients when compared to patients with HIV-1. HIV-2 transmission is also less efficient^6^,^7^ and has a lower viral load and a slower decline in CD4+ T-cell counts observed during asymptomatic infection^8^,^9^,^10^,^11^. Over 80% (86–95%) of HIV-2 patients can be considered as long-term non progressors (LTNP), while at most 15% (5–15%) LTNPs are observed in most HIV-1 cohort studies^8^,^11^,^12^,^13^. The contributing factors to these differences remain largely unknown but comparative studies between HIV-1 and HIV-2 infections have led to several hypotheses. Thus, it has been proposed that lower rates of T-cell activation in HIV-2 infected patients^14^,^15^,^16^, better immune control^11^,^17^,^18^, lower mutation rates, and replication capacity of HIV-2^8^,^19^,^20^ could account for these differences. Yet, in at least 10% of HIV-2 infected patients the profile of infection is similar to that observed with HIV-1. Therefore, we reasoned that the comparison of isolates from HIV-2 rapid progressors (RP; high viral load, CDC stage C) versus HIV-2 LTNP (undetectable viral load, CDC stage A) might be more informative than the overall comparison of the two types of viruses.

After entering in a new host cell, retroviruses must hijack cellular host proteins to complete many aspects of their viral replication cycle while counteracting cellular restriction factors. These are components of the innate immune response^21^ such as the apolipoprotein B mRNA-editing enzyme-catalytic polypeptide-like 3G (APOBEC3G)^22^ BST-2/CD317/tetherin^23^,^24^, TRIM5alpha proteins^25^, the SAM domain and HD domain-containing protein 1 (SAMHD1)^26^,^27^ or the myxovirus resistance 2 (MX2)^28^,^29^. HIV-2 has been transmitted to humans from sooty mangabeys, a lower monkey species compared to chimpanzees. Therefore it has been evoked that HIV-2 may be more susceptible to innate human restriction factors than HIV-1. A possible correlation between variations in the sequences of HIV-2 strains and the capacity to replicate in humans, associated with clinical progression to AIDS has been previously envisaged. For example, a yet unidentified factor, called Lv2, may restrict certain HIV-2 strains after virus entry, but not HIV-1^30^,^31^, and more recently, the RNA-associated early-stage anti-viral factor (REAF) has been shown to inhibit HIV-1 and HIV-2 to a higher level^32^.

After retroviral entry, the viral capsid (CA) which plays an important role during the early steps of the viral cycle, i.e. uncoating, reverse transcription, nuclear import and integration^33^, becomes an important target to host cellular factors, and influences innate responses such as DNA sensing mediated by cGAS/PQBP1 through the STING pathway in cells of myeloid origin^34^,^35^.

One of the restriction factors recognizing the incoming capsid is the primate TRIM5alpha factor, a cellular E3 ubiquitin ligase that restricts infection and promotes innate immune signaling in response to retroviral infection^36^,^37^,^38^. TRIM5alpha blocks viral infection before reverse transcription and is species-specific^25^. Several single nucleotide polymorphisms (SNPs) in human TRIM5alpha have been found to modulate HIV-1 infection, notably in regard of the time taken to disease progression^39^ and its correlation with different restriction potentials^40^,^41^,^42^. However, association between human TRIM5alpha variation and HIV-2 has not yet been described. Overall, HIV-2 is more sensitive to restriction by TRIM5alpha than HIV-1^43^,^44^,^45^,^46^ and variations in sensitivity have been linked to specific proline residues within the HIV-2 capsids^47^,^48^. However, there is no clear correlation between *ex vivo* susceptibility to TRIM5alpha and viral loads in patients^45^.

In addition to differences in sensitivity of HIV-2 to the already identified innate cellular factors when compared to HIV-1, is that HIV-2 could exploit the recruitment of alternative host factors for optimal infection. Interestingly, cyclophilin A (CypA) that is encoded by the *peptidyl prolyl isomerase A* gene (*PPIA*), is an essential cofactor of the early steps of HIV-1 infection in human cells^49^,^50^,^51^. While CypA interacts specifically with HIV-1 CA^52^,^53^, its interaction with HIV-2 CA is not clear^54^,^55^,^56^.

In a previous study, we have shown that both HIV-1 and HIV-2 prototypes exploit the same pathway to enter the nucleus, i.e. a dependence on the CypA, Nup358 and Nup153 factors^57^, which may reflect an adaptation to human cells for high replication. However, little is known on the overall susceptibility of different HIV-2 primary isolates to host factors and progression to disease. Since CA determinants play an essential role in the early stages of viral replication, the aim of our study was to evaluate the interactions of HIV-2 p26/CA determinants, with CA-interacting cellular proteins, including cytoplasmic TRIM5 proteins, CypA and nucleopore-associated Nup358 and Nup153. The CA variants were derived from HIV-2 primary isolates obtained from either asymptomatic or symptomatic patients that were included in the French ANRS Cohort CO5.

## Results

In order to investigate the susceptibility to different intracellular host factors of CA that are present in primary isolates from viremic or aviremic HIV-2 patients, we amplified and sequenced the matrix-capsid (MACA) regions from DNA extracted PBMCs of 18 HIV-2 infected participants included in the French ANRS Cohort CO5 (10 symptomatic and 8 asymptomatic). All of the HIV-2 sequences that were obtained belonged to the epidemic groups A or B. We then chose to further assess the sequences that differed in CA at specific positions (proline residues at positions 119, 159 and 178) that have previously been described as differentiating between low and high viral load individuals^47^, as well as to play a role in TRIM5alpha restriction. Sequences found in RP (high viral load, severely symptomatic) or LTNP (undetectable viral load, asymptomatic) patients were closely related to each other, including in the CypA-binding loop (Fig. S1). Although the HIV-2_ROD_ prototype has a “PPP” motif, this motif was not found in our patients. Nevertheless, all of the RP had a P at position 178. After analysis of multiple sequences from primary isolates, we noted that “APP” was the most frequent motif. We therefore selected the CA from two patients with “APP” to test whether any other variations in CA sequence could account for phenotypical differences. The virological and clinical characteristics of these HIV-2 patients are summarized in Table 1.

In order to functionally address these points, we introduced different versions of HIV-2 MACA within a *gag-pol* SIVmac expression vector using a previously validated strategy^57^. The resulting chimeric were used to produce single-round infection viruses. Upon transfection of HEK-293T cells, we produced VSV-G pseudotyped eGFP or Crimson-expressing chimeric HIV-2 MACA from selected donors along with a control sequence harboring MACA of HIV-2_ROD_. Efficient proteolytic cleavage of p57Gag was assessed on viral pellets by CA immunoblotting using an anti-HIV-2 serum (Fig. S2A) and virion infectivity was tested in a Mus dunni cell line, which do not have TRIM5alpha mediated restriction (Fig. S2B). The chimera #13 from a LTNP donor that includes a “GPT” motif had a low titer and was therefore not used in further experiments. Interestingly, all chimeras derived from the HIV-2 patients that have high viral load led to higher titers when compared to chimeras derived from individuals with low viral load (Fig. S2B).

### Hu-TRIM5alpha does not efficiently restrict any of the HIV-2 primary CA isolates

**Figure 1:**
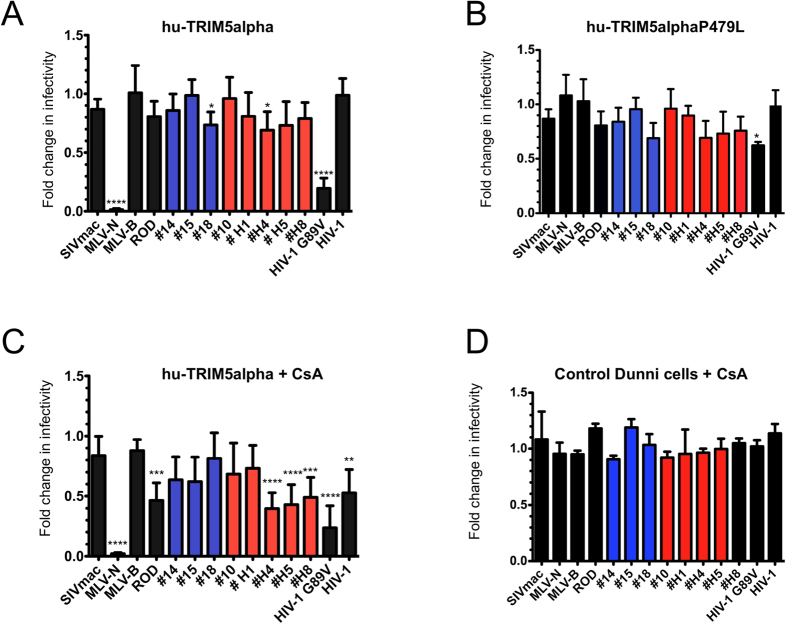
Mild restriction of HIV-2 infection by hu-TRIM5alpha proteins is increased by CsA treatment. Control dunni cells or dunni cells stably expressing TRIM5alpha WT- (**A**) or hu-TRIM5P479L (**B**) proteins were infected by VSV-G pseudotyped eGFP-expressing virions harboring either wt MLV-N, MLV-B, SIVmac, HIV-1 or HIV-2_ROD_ CA, or the HIV-1G89V CA mutant, as controls; or the different chimeric SIVmac/HIV-2 CAs. HIV-2 chimeric viruses from non progressor patients are shown in blue and those from progressor patients in red. (**C**) Restriction activity in dunni cells expressing WT hu-TRIM5alpha in the presence of Cyclosporin A (CsA). (**D**) Restriction activity in control dunni cells in the presence of CsA. Infected cells were counted by flow cytometry. Percentages of infected TRIM5alpha-negative control cells obtained with CA from LTNP or RP ranged between 14–30% and 50–82%, respectively. Fold changes in infectivity were calculated as the ratio of percentage of eGFP-positive TRIM5alpha-positive cells to that of eGFP-positive infected control dunni cells (A and B), the ratio between eGFP-positive TRIM5alpha-positive cells in the presence of CsA to the eGFP-positive infected control dunni cells (**C**), and the ratio between eGFP-positive control dunni cells in the presence of CsA to that of eGFP-positive control dunni cells without CsA. (**D**) Results are from at least three independent experiments. One-way ANOVA with Tukey’s multiple comparisons test was used to assess significance. SEM and P-values are indicated (*P < 0.05; **P < 0.01 ***P < 0.001; ****P < 0.0001).

SNP in genes involved in innate immunity, such as TRIM5alpha, may contribute to the difference in human susceptibility to HIV-1 infection and subsequent disease progression^39^,^41^,^58^. Since HIV-2 is more susceptible to TRIM5alpha than HIV-1, we wondered whether a correlation could be established between TRIM5 polymorphisms and susceptibility to HIV-2 infection and disease progression in our patients. We therefore amplified and sequenced the exon 8 of TRIM5alpha, corresponding to the PRY/SPRY domain that is required for capsid restriction^59^,^60^ from the genomic DNA of the 18 donors. We found that one patient (#H1) had a heterozygous SNP with a substitution of a proline (P) for leucine (L) in the V4 region at position 479. We tested whether this SNP introduced a change in the susceptibility of CA from HIV-2 primary isolates, as compared to the reference hu-TRIM5alpha sequence. Dunni cells stably expressing WT hu-TRIM5alpha or the P479L allele were challenged with single-round VSV-G pseudotyped eGFP virions. Stable expression of hu-TRIM5alpha in these cells was verified by western blot analysis against its HA-epitope tag (Fig. S2C). As shown in Fig. 1A and B, the overall restriction profiles of WT and P479L hu-TRIM5alpha to all the HIV-2 CA were similar, and no specific susceptibility to TRIM5alpha WT or variant was observed for #H1 CA. This indicates that this mutation does not seem to play a role in the patient disease progression. This is in agreement with the fact that a P479L mutation found in one HIV-1 cohort study was not linked to infection or AIDS progression^41^. As observed by others^45^, CA from HIV-2 primary isolates have a greater susceptibility to hu-TRIM5alpha than to HIV-1. This enhanced HIV-2 susceptibility is present regardless of the disease status or the motifs in the capsid sequences (Table 1). Indeed, CA sequences as present in RP or LTNP patients were mildly restricted by hu-TRIM5alpha (ratio 0.7 to 0.8) while #15 CA (LTNP and motif “PPA”) and #10 CA (RP and motif “VPP”) were totally insensitive (Fig. 1A and B). However, the infectivity of the positive control CA (N-MLV) was highly reduced (ratio 0.01, p < 0.001) by WT hu-TRIM5alpha, but remained unaffected by P479L hu-TRIM5alpha to the same extent as B-MLV or SIVmac controls, which are resistant to restriction (Fig. 1A and B). Notably, the restriction activity against HIV-1-G89V, a mutant that lacks the ability to bind CypA (ratio 0.2, p < 0.001), which has already been observed by us and others^43^,^57^,^61^, was partially lifted (ratio 0.6) by the TRIM5alpha P479L mutation (Fig. 1A and B). This finding argues in favor of a direct interaction between CA and TRIM5alpha.

### Inhibition of CypA binding by addition of CsA results in the restriction of virions harboring CA derived from HIV-2 primary isolates

CypA has been shown to bind HIV-1 CA and play a key role during HIV-1 infection and replication processes^62^. This binding has been shown to influence HIV-1 CA susceptibility to restriction factors^63^. Several lentiviruses bind CypA with high affinity, unlike SIVmac. Since the interaction of HIV-2 CA with CypA is still under debate^55^,^56^, we tested whether the susceptibility of some CA from HIV-2 primary isolates to hu-TRIM5alpha were linked to CypA binding. For this purpose, we monitored single-round infectivity of virions that harbor different HIV-2 CA in the presence of cyclosporin A (CsA), a competitive inhibitor to CypA binding. As expected, addition of CsA had no effect on N and B-tropic MLV, as well as on SIVmac infections. HIV-1 infection was reduced twofold, in agreement with several studies that showed an interaction between HIV-1 CA and CypA (Fig. 1C). Infectivity of the HIV-1 G89V mutant that is unable to bind CypA, remained reduced as previously observed. Interestingly, all HIV-2 chimeras (including HIV-2_ROD_,) were more sensitive to hu-TRIM5alpha in the presence of CsA, although the restriction levels vary with the different primary isolate CA (Fig. 1C). Interestingly, CsA effect appeared to be dependent on stable expression of TRIM5alpha, as no effect was observed in dunni control cells (Fig. 1D) as compared to Fig. 1C. Altogether, these results unveiled a CypA dependence for HIV-2 CA mediated infection, similar to that of HIV-1.

### Susceptibility of HIV-2 CA from primary isolates to TRIMCyp

**Figure 2:**
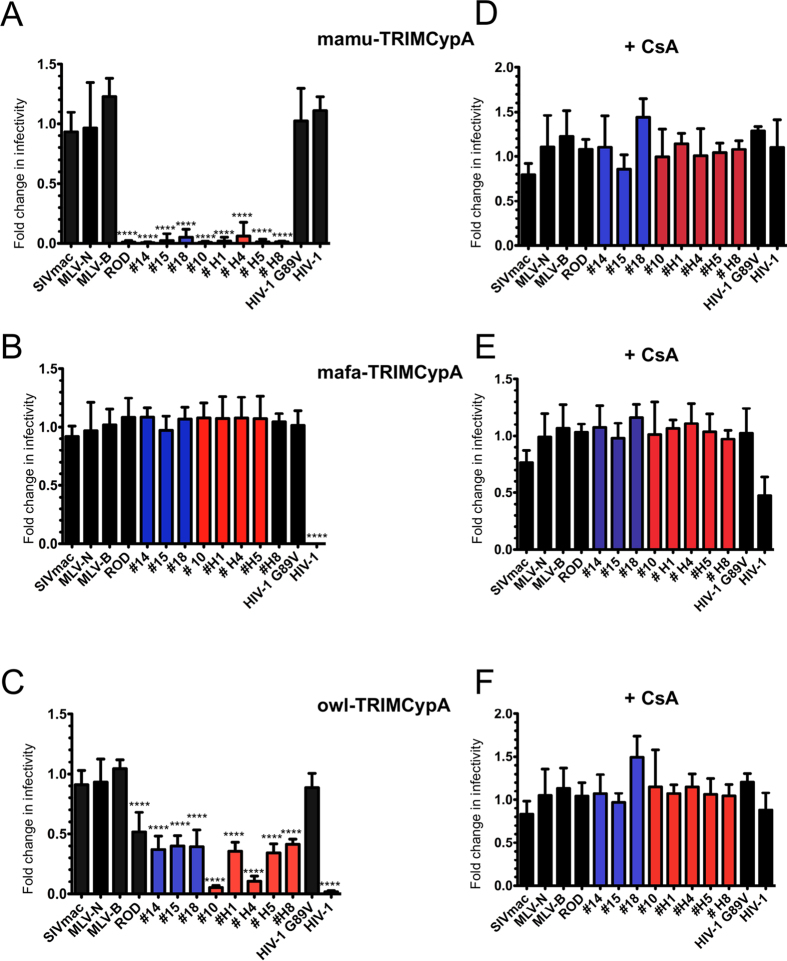
Owl-TRIMCypA and mamu-TRIMCypA restrict HIV-2 infection. Control dunni cells or dunni cells stably expressing either mamu-TRIMCypA (**A**), mafa-TRIMCypA (**B**) or owl-TRIM5CypA (**C**) were infected by the VSV-G pseudotyped eGFP-expressing virions described in Fig. 1. Restriction activity in the presence of CsA (**D** to **F**) was expressed as fold change in infectivity and calculated as in Fig. 1. Results are from at least three independent experiments. One-way ANOVA with Tukey’s multiple comparisons test was used to assess significance. SEM and P-values are indicated (****P < 0.0001).

Given that we have observed that all CA derived from HIV-2 primary isolates, as well as HIV-2_ROD_ CA, were sensitive to the presence of CsA, we investigated whether HIV-2 CA from viremic or aviremic patients differed in their ability to bind to CypA. To this goal, we took advantage of TRIMCyp fusion proteins that are naturally present in several species of monkeys. Owl-TRIMCypA was described in owl monkeys^64^,^65^, while mafa-TRIMCypA and mamu-TRIMCypA proteins were found in different macaque species^66^,^67^. Mamu-TRIMCypA has been shown to block HIV-2 infection while owl-TRIMCypA and mafa-TRIMCypA proteins block HIV-1 infection^67^. We thus tested the infectivity of different chimeric HIV-2 Gag in dunni cells stably expressing mamu-TRIMCypA, mafa-TRIMCypA or owl-TRIMCypA proteins. TRIMCypA expressions were confirmed by western blot using an anti-HA mAb (Fig. S2C). Consistent with a previous report^67^, we also observed that HIV-2_ROD_ was strongly restricted by mamu-TRIMCypA while SIVmac, N and B-MLV, HIV-1 and the G89V mutant were not (Fig. 2A). Strikingly, all CA found in HIV-2 primary isolates were restricted as efficiently as HIV-2_ROD_ CA (Fig. 2A). This is consistent with the fact that all the sequences of the CypA binding loop of the tested HIV-2 CA were closely related to each other with the deletion of an alanine residue at position 88, which is present in HIV-1 CA^56^. CypA-dependent susceptibility was confirmed by restoration of infectivity in the presence of CsA (Fig. 2D). As reported by others^68^, expression of mafa-TRIMCypA severely restricted HIV-1 infection while the HIV-1 G89V mutant and HIV-2_ROD_ escaped restriction (Fig. 2B). All HIV-2 CA from primary isolates that we tested were also insensitive to mafa-TRIMCypA with no effect from CsA, which lifted HIV-1 infectivity as expected (Fig. 2E). Surprisingly, although owl-TRIMCypA exhibited a restriction profile similar to that of mafa-TRIMCypA, we documented significant differences in the levels of susceptibility of HIV-2 primary isolate CA to owl-TRIMCypA (Fig. 2C). Thus, we could distinguish two groups of restricted phenotypes. A group of CA, including that of HIV-2_ROD_, which were partially restricted (ranging from 2.5 to 3.3 fold). This group included isolates from both LTNP (#14,#15 and #18) and RP individuals (#H1,#H5 and #H8). A second group of CA that were robustly restricted (from 10 to 15 fold), that included isolates #10 and #H4, both derived from RP individuals (Fig. 2C). These distinctive levels of restriction were also CypA dependent as all were abrogated upon CsA treatment (Fig. 2F). Intriguingly, despite their identical CypA binding loop sequences, CA #H4 and #H5 were distinctly sensitive to owl-TRIMCypA restriction, which unveiled the role of other determinants in CA that modulate CypA-dependent restriction. Nevertheless, this difference did not seem to explain progression to AIDS.

### HIV-2 infection dependence to CypA

**Figure 3:**
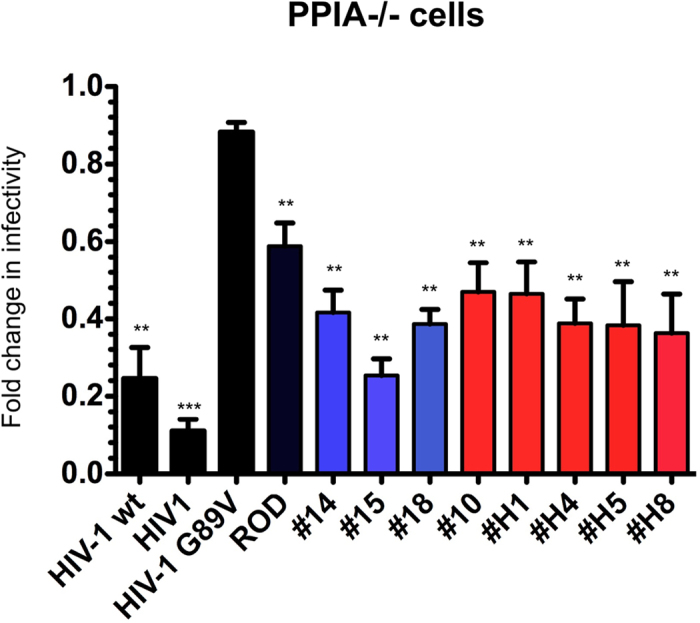
Optimal infection by HIV-2 isolates requires CypA. Jurkat cells or Jurkat PPIA^−/−^ cells were infected by VSV-G pseudotyped eGFP-expressing virions harboring either HIV-1 wt, HIV-1 G89V mutant, or HIV-2_ROD_ CA as controls, or the chimeric SIVmac/HIV-1 CA (HIV-1) or SIVmac/HIV-2 CA. HIV-2 chimeric viruses from non progressor patients are shown in blue and those from progressor patients in red. Infection levels were determined as in Fig. 1, as the ratio of percentage of PPIA−/− Jurkat cells that were GFP-positive to GFP-positive control Jurkat cells. Results are from at least two independent experiments. Unpaired two-tailed Student’s t-test was used to assess significance. SEM and P-values are indicated (**P < 0.01; ***P < 0.001).

Since the results presented above unveiled a potential role of CypA in HIV-2 infection, we tested whether depletion of CypA in human cells influenced HIV-2 early-steps of infection. For this purpose, we compared infection levels of all HIV-2 chimeras in parental or PPIA^−/−^ Jurkat cells. In the latter cells, both alleles of the CypA-encoding *PPIA* have been inactivated^69^. While infectivity of the HIV-1 G89V mutant was sustained (ratio 0.9), all HIV-2 CA tested were significantly impacted by the lack of CypA, although to a lower extent than WT HIV-1 CA (Fig. 3). Of note, infection by chimera #15 that was isolated from an LTNP donor, was as affected that HIV-1 by the lack of CypA. These results strengthen our observation that there is a crucial and general role for CypA binding in HIV-2 infections of human cells.

### CA from HIV-2 primary isolates is the determinants for infectivity dependency on Nup358 and Nup153

**Figure 4:**
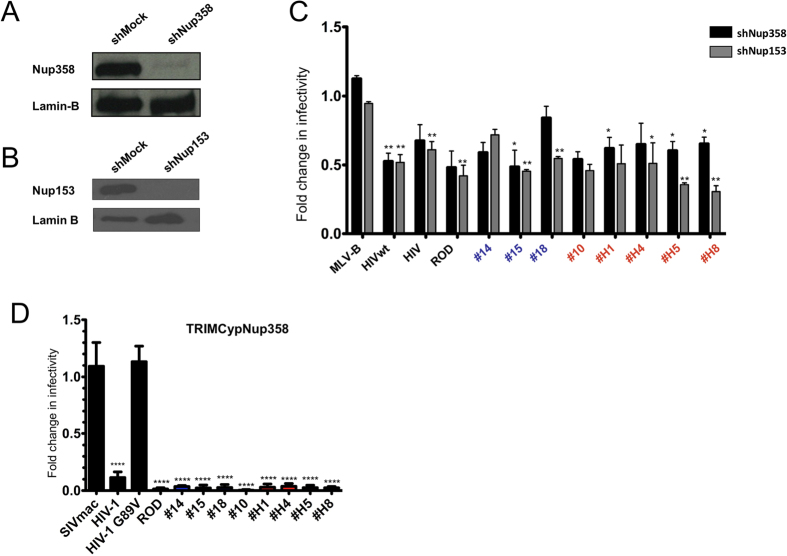
Modulation of Nup358 and Nup153 expression, impact on infection by HIV-2 isolates and CA binding. Levels of expression of Nup358 (**A**), Nup153 (**B**), or lamin-B in control Jurkat cells that stably express shRNA against either a non-vertebrate gene (shMock), *Nup358* or *Nup153*. (**C**) Cells in A and B were challenged with MLV-N or HIV-1wt CA as controls, and recombinant viruses carrying multiple HIV-1 or HIV-2 CA sequences. Infection levels were determined as in Fig. 1, as the ratio of the percentage of Crimson+ and Nup358 KO (black bars) or Nup153 KO (grey bars) Jurkat cells, to Crimson + shMock control Jurkat cells. The names of the HIV-2 chimeric viruses from non progressor patients are shown in blue and those from progressor patients in red. Unpaired two-tailed Student’s t-test was used to assess significance. SEM and P-values are indicated (*P < 0.05; **P < 0.01). (**D**) Control dunni cells and dunni cells expressing TRIMNup358Cyp were infected with the virions described above, MLV virions excluded. Restriction activity in dunni cells that were GFP-TRIMCypNup358 positive was calculated as described above in comparison with GFP-positive control dunni cells. Chimeric viruses from non progressor or progressor patients are as above. Results are from at least three independent experiments. Unpaired two-tailed Student’s t-test was used to assess significance. SEM and P-values are indicated (***P < 0.001).

In contrast to other retroviruses, lentiviruses have the ability to productively infect non-dividing cells, and it has been shown that this ability is conferred by the CA determinants^70^,^71^. In a previous study we found that, like HIV-1, HIV-2_ROD_ prototypic infection was decreased upon depletion of Nup358 or Nup153, suggesting a common pathway to enter the nucleus^57^. Here, we examined whether the CA from primary HIV-2 isolates had similar dependence to these nucleoporins. We transduced Jurkat cells with a lentiviral vector expressing control shRNA (mock) or a shRNA targeting either Nup358 or Nup153. Specific depletion of the proteins was verified by immunoblotting of whole cells extracts from stable Nup358 or Nup153-depleted Jurkat cells (Fig. 4A and B). As expected, upon knockdown of Nup358, MLV-B infection was unaffected while HIV-1 and HIV-2_ROD_ infections were significantly inhibited when compared to control Jurkat cells (Fig. 4C). Infectivity of CA determinants from HIV-2 primary isolates from either LTNP patients or RP donors were also inhibited (ratio 0.45 to 0.6) (Fig. 4C). However, the dependence of CA from LTNP HIV-2 patient #18 to Nup358 was marginal (ratio 0.85) (Fig. 4C).

Nup358 has a C-terminal domain that is homologous to CypA, which has been shown to bind HIV-1 CA^72^,^73^. We previously generated a pLXSN retroviral vector encoding a synthetic TRIMCypNup358 protein that comprises the RBCC domain of owl-TRIMCypA fused to the Nup358CypA domain. We derived dunni cells that stably expressed the chimeric TRIMCypNup358 protein, and confirmed expression of the fusion protein by western blot with an anti-HA antibody (Fig. S2C). Strikingly, in contrast to SIVmac and HIV-1 G89V, HIV-1 and HIV-2 prototypes and all the CA from HIV-2 primary isolates were strongly restricted by TRIMCypNup358, thus demonstrating a direct interaction of CA with the Nup358 CypA-like domain (Fig. 4D). Although the CypA-binding loop of HIV-2 CA is quite different from that of HIV-1, our present finding is in accord with the fact that the Nup358 CypA-like domain is involved in the interaction of the latter with HIV-1 CA^74^,^75^. Of interest, this contrasted with our previous study wherein we found no correlation between sensitivity of circulating SIV isolates to Nup358 depletion and restriction by TRIMCypNup358^57^.

While the MLV-B control is not affected by stable Nup153 depletion in Jurkat cells, this depletion significantly and specifically altered infectivity of HIV-1 and HIV-2_ROD_ as well as that of all primary HIV-2 isolates (ratio 0.25 to 0.65) (Fig. 4C).

## Discussion

HIV-1 and HIV-2, which arose from distinct SIV zoonotic transmissions, present several differences in terms of originating species, geographical distribution, replication, transmission, and progression to AIDS^12^. Furthermore, while HIV-1 patients that meet the criteria of LTNP are rare, most HIV-2 infected patients exhibit LTNP virological and clinical profiles. Nevertheless, as some HIV-2 infected individuals will develop AIDS, few data are available on interactions of CA with host factors that would characterize this stage. In this study, we evaluated the ability of HIV-2 primary isolate capsids to interact with host cellular factors known to negatively or positively modulate HIV-1 infection. For this purpose, we derived and compared CA from individuals with high viral load that were rapidly progressing to AIDS versus those from HIV-2 infected patients with undetectable viral load and considered as LTNPs.

As previously reported by others, we found that HIV-2 CA from primary isolates were more susceptible to hu-TRIM5alpha than HIV-1 CA. The role of proline residues at CA positions 119, 159 and 178 has been associated with highest hu-TRIM5alpha susceptibility for HIV-2_ROD_ CA^45^,^47^,^48^. In contrast, we did not find any obvious correlation between the “PPP” pattern found in the primary isolate CA and viral load, progression to disease, or susceptibility to hu-TRIM5alpha, as tested *in vitro*. Furthermore, although all CA motifs found in rapid progressors harbored a P178 these CA were marginally or not detectably restricted by hu-TRIM5alpha. Therefore, our observations argued in favor of a key role for other CA residues with regard to susceptibility of HIV-2 to hu-TRIM5alpha.

Cohort studies have suggested that some hu-TRIM5alpha SNPs are associated with a protective effect against HIV-1 infection^39^,^41^. However, to our knowledge, no population study has reported a correlation between hu-TRIM5alpha polymorphism and HIV-2 susceptibility *in vivo*. Although we found that the TRIM5alpha P479L SNP, located in the PRY/SPRY domain of a RP patient, did not change the restriction pattern towards HIV-2 CA, it would remain of interest to evaluate larger cohorts of HIV-2 patients for the possible contribution of hu-TRIM5alpha polymorphisms to HIV-2 replication.

It has been reported that HIV-2 does not require host CypA for efficient replication in human cells^54^. Even if a weak binding between HIV-2 CA and CypA has been detected in a previous report^56^, the interaction of the HIV-2 CA with CypA remains debated. We found that addition of CsA in mouse dunni cells that over-express hu-TRIM5alpha enhanced the restriction activity against all tested CA from HIV-2 primary isolates. CsA is known to bind CypA and to inhibit its interaction with other proteins. Since CsA enhanced hu-TRIM5alpha-mediated restriction of all CA from HIV-2 primary isolates, this indicated that these isolates required CypA for optimal replication. As the amino acid sequences of mouse and human CypA are 98% identical, it is unlikely that CypA differences between species could play a role in this phenotype.

In this context, we evaluated whether CA HIV-2 primary isolates differed in their potential binding to hu-CypA and subsequent infectivity. The efficient restriction observed with the natural owl-TRIMCypA on all HIV-2 primary isolate CA strongly suggests that all CA from primary isolates can interact with CypA. We found that these interactions resulted in a drop of the infectivity mediated by the HIV-2 CA, even in the case of two HIV-2 CA that were derived from rapid progressors, reaching the same levels of restriction as that seen with HIV-1 CA. This was confirmed by the reduced infectivity of HIV-2_ROD_ prototype and all the HIV-2 primary isolate CA we tested in Jurkat PPIA^−/−^ cells when compared to Jurkat cells. Nevertheless, no difference in CypA-CA interactions could be established between CA from rapid progressors versus LTNPs. Therefore, in contradiction to some previous studies, our results underline the fact that HIV-2, like HIV-1, is dependent on CypA for optimal infection in human cells. Assessing whether more subtle differences in the binding affinity of HIV-2 CA to CypA play a role in disease progression will require more highly sensitive measurements.

Indeed, it was recently reported that the affinity of HIV CA for CypA affects sensing of viral DNA mediated by cGas in monocyte-derived dendritic cells^34^ and may contribute to the differential pathogenesis of HIV-2 in LTNPs versus RPs.

CypA, Nup153 and Nup358 interact with CA and have been described to be part of a pathway that mediates HIV-1 nuclear import^73^,^75^,^76^,^77^. In a previous study, we have shown that HIV-2_ROD_ exploits the same pathway, while circulating SIV isolates can use alternative pathways to enter the nucleus^57^. Here, we tested the status of HIV-2 primary isolates with regard to Nup153 or Nup358 interaction, and found that all CA from HIV-2 primary isolates were sensitive to the absence of Nup358. Although infectivity of HIV-2 CA derived from #18 LTNP seemed less affected by Nup358 depletion, all HIV-2 infections were strongly restricted by the artificial TRIMCypNup358 fusion protein. This demonstrated the general ability of HIV-2 primary isolate CA to directly bind to Nup358 Cyp-like domain, as previously described for HIV-1 and for HIV-2 prototypes^57^,^73^. Binding of HIV-1 CA to the Cyp-like domain of Nup358 depends on residue P90^75^. Although the CypA-binding loop of HIV-2 CA is shorter than that of HIV-1 CA, the corresponding GP motif is maintained in all HIV-2 primary isolate CA. It is therefore tempting to speculate that HIV-2 CA may be isomerized since Nup358 is required for its infection. However, despite the fact that FIV CA binds Nup358, the CA does not use Nup358 as co-factor and is not propyl isomerized by the Cyp-like domain of Nup358^75^.

We found that all HIV-2 infections were also decreased upon Nup153 depletion. Others have previously shown that binding of HIV-2_ROD_ CA and HIV-1 CA to Nup153 is required for optimal infection, especially in association to four residues, N56, Q66, R69, N73 in HIV-2_ROD_ CA^77^. Accordingly, all four residues were conserved in HIV-2 primary isolates tested here. We further observed that this dependence of HIV-2 primary isolates and HIV-1 on the same cellular co-factors also extended to TNPO3 (data not shown); of note, TNPO3 is a karyopherin known to transport SR family proteins, which has been also implicated in the Nup358/Nup153 pathway^78^,^79^. As the decrease of infectivity of HIV-1 upon TNPO3 depletion has been shown to be dependent on cleavage and polyadenylation specific factor 6 (CPSF6)^80^, we hypothesized that the proposed model for HIV-1 nuclear entry could also be applied to HIV-2 nuclear entry^74^,^81^. Interestingly, both SIVcpz and SIVsm adaptation to human cells seems to exploit the same co-factors and pathway for nuclear import. Although these cellular factors appeared to be recruited by the two epidemic groups A and B, it may not be the case for other groups that may have had fewer, if not a single, transmission events.

We found that sensitivity to the cellular host factors we tested and HIV-2 disease progression remained dissociated. However, here we showed that CypA dependence is a common trait of all HIV-2 CA from primary isolates. Nevertheless, while requiring CypA, these isolates may still differ in their requirements for other factors that interact with HIV-1 CA. Such potentially distinctive factors include the recently identified protein SUN2 that seems essential for HIV-1 infection of CD4^+^ T cells^82^,^83^, or MX2, which has been reported to inhibit HIV-1 infection^28^,^84^. Assessing interaction of HIV-2 primary isolate CA with the latter would be of particular interest since it is induced by IFN and since HIV-2 has been shown to be less resistant than HIV-1 to IFN response^85^.

## Methods

### PCR amplification and sequencing of the TRIM5alpha PRY/SPRY region

Genomic DNA was extracted from PBMC from 18 patients from the French ANRS CO5 HIV-2 cohort using the QIAamp blood kit (Qiagen), according to the manufacturer’s instructions. Written informed consents were obtained for all patients at the time of inclusion in the cohort. All experiments were performed in accordance with relevant guidelines and regulations. This study was approved by the ethics committee (CPP Ile-de-France IX). For analysis of variations in the TRIM5 exon8, DNA samples were amplified by PCR using the Expand High Fidelity PCR System (Roche Diagnostics) with the specific primers (5′-GGTTCCTCCCAGTTTTCTCTCAAG-3′) and (5′-GAAGGGGCTGAGTGTGTAAGAAGG-3′). The PCR products were purified using the PCR clean-up Gel Extraction kit (Macherey Nagel) according to manufacturer’s protocol and used for direct Sanger sequencing. DNA sequencing chromatograms were examined visually to detect heterozygous SNPs at each position using Geneious Pro software and amino acid sequences were aligned using Clustal W.

### Generation of cells stably expressing TRIM5 variants

The full-length cDNA from human TRIM5alpha was obtained from RT-PCR amplification of HeLa cells RNA. The TRIM5alpha P479L was obtained by recombinant PCR by exchanging the human TRIM5alpha exon 8 with the corresponding mutated allele from patient #H1. The full-length owl-TRIMcypA cDNA was PCR amplified from plasmid pMIG-TRIMCyp obtained from the NIH AIDS reagents program^65^. Mafa-TRIMCypA and mamu-TRIMCypA were kind gifts of Dr. Greg Towers^68^,^86^. The TRIM-CypNup358 was obtained by recombinant PCR as previously described^57^. All PCR products were cloned into a retroviral vector containing two C-terminus hemagglutinin (HA) tags derived from pLXSN (Clontech). HEK-293T cells were then independently co-transfected with the pLXSN-based retroviral vectors encoding the various HA-tagged TRIM5 variants along with plasmids expressing MLV Gag-Pol and the vesicular stomatitis virus (VSV) G envelope glycoprotein (pCSIG)^87^. Forty-eight hours after transfection, the retroviral supernatants were harvested and used to transduce dunni cells grown in the presence of G418 at 2 mg/ml (Invivogen) to select stable expression of TRIM5.

### Chimeric gag-pol SIVmac expression plasmids

The HIV-2 *gag* matrix-capsid fragments (MACA) were PCR amplified from DNA extracted from pelleted PBMCs from 18 infected patients of the French ANRS CO5 HIV-2 cohort. The MACA PCR products were first subcloned into a pUC19 vector containing a fragment of a *gag-pol* SIVmac251 expression plasmid (pAd-SIV4)^88^ as previously described^57^. The most represented CA sequence obtained from the patient was then introduced into the pAd-SIV4 vector to obtain chimeric *gag-pol* SIVmac expression plasmids. The HIV-1 and HIV-1G89V *gag* fragments were PCR amplified from the p8.91 and p8.91G89V, *gag-pol* expression plasmids, respectively^89^,^90^.

### Cell culture

HEK-293T and Mus Dunni (dunni) cells^91^ were cultivated in Dulbecco modified Eagle medium supplemented with 10% fetal bovine serum (FBS), non-essential amino acids and penicillin 100 U/ml and streptomycin 0.1 mg/ml at 37 °C and 5% CO2-air atmosphere. Jurkat cells were grown in RPMI containing 10% FBS. Jurkat T-Cells CypA−/− were obtained through the NIH AIDS Reagent Program, Division of AIDS, NIAID, from Drs. D. Braaten and J. Luban^69^.

Infection with VSV-G pseudotyped viruses expressing green fluorescent protein (GFP). HIV-1, SIVmac and chimeric HIV-2 vectors carrying the green fluorescent protein (GFP)-reporter gene were generated by co-transfecting HEK-293T cells with three expression vectors; (i) a Gag-Pol expression vector (p8.91 N∆SB for HIV and pAd-SIV4 for SIVmac and chimeric SIV) (ii) the VSV-G envelope glycoprotein expression vector (pCSI-G), which allows efficient virion fusion into the mammalian cell lines, and (iii) a vector providing a packageable GFP-containing retroviral RNA genomes: pSIN CSGW^92^ for HIV genomes and GAE-SFFV-GFP-WPRE for SIV genomes^88^. Virus-containing culture supernatant was harvested 48h after transfection and filtered through a 0.45 μm-pore-size filter before immediate storage at −80 °C until use.

Viral stock infectious titers were determined via GFP expression by flow cytometry 48h after viral challenge of non-restricting dunni cells. All infections were performed in 96-well plates, wherein 2 × 10^4^ cells were incubated with two-fold serial dilutions of the challenging virus. Polybrene was used in all transductions at a concentration of 4μg/ml. When required, Cyclosporin-A (Sigma-Aldrich) was added at a concentration of 5μg/ml. Cells were then incubated at 37 °C/5%CO2. GFP-positive cells were enumerated 48h later on a FACSCalibur flow cytometer (Becton Dickinson) after collection of at least 10,000 events. Data analyses were performed using FlowJo 7.6.1 software (Tree Star).

### shRNA transduction and infection with VSV-G pseudotyped viruses expressing E2-Crimson fluorescent protein

The sequences of shRNA pLK0.1-GFP plasmid for Nup358 were as described in^69^ with a modification to the hairpin loop by adding the nucleotides of the human miR-30 loop. The sequence of shRNA pLK0.1-GFP plasmid Nup153 was from Sigma-Aldrich (SHCLNG-NM_005124). Virions were produced in a similar way as HIV-1 VSV-G pseudotyped viruses. After transduction, Jurkat cells were sorted twice for GFP-positive populations using a BD FACSAria cell sorter (Becton Dickinson). GFP Jurkat cells were then infected with VSV-G-pseudotyped Crimson reporter viruses.

### Western blotting

Dunni cells stably expressing HA-tagged TRIM5 variants were lysed in 100 mM NaCl, 50 mM Tris (pH 7.5) and 1% Triton X-100 containing protease inhibitors (Sigma-Aldrich). Proteins were separated on a 10% acrylamide gel, transferred onto PVDF membrane and probed with a rat anti-HA antibody (3F10, Roche Applied Science). A peroxidase-conjugated goat anti-rat (SouthernBiotech) was used as secondary antibody. Loading was controlled by probing with a β-actin antibody. For CA detection, conditioned cell-free supernatants of transfected HEK-293T cells were pelleted through a 20% sucrose layer in TEN buffer (10 mM Tris.HCl pH7.5; 100 mM NaCl; 1 mM EDTA) at 25000 rpm for 2h30 at 4 °C in a SW 40 Ti rotor. Pelleted viruses were probed for CA content using sera from HIV-2 infected patients and anti-human-IgG-HRP IgG (Sigma-Aldrich) as primary and secondary antibodies, respectively.

Protein depletion by shRNA in Jurkat cells was monitored by immunoblotting using a rabbit polyclonal antibody against Nup358/RanBP2 (ab64276, Abcam) and mouse Nup153 antibodies (ab24700, Abcam) with a peroxidase-conjugated goat anti-rabbit and anti-mouse as secondary antibodies, respectively (Sigma-Aldrich). Lamin-B was used as a loading control.

## Additional Information

**How to cite this article:** Mamede, J. I. *et al*. Cyclophilins and nucleoporins are required for infection mediated by capsids from circulating HIV-2 primary isolates. *Sci. Rep.* **7**, 45214; doi: 10.1038/srep45214 (2017).

**Publisher's note:** Springer Nature remains neutral with regard to jurisdictional claims in published maps and institutional affiliations.

## Supplementary Material

Supplementary Information

## Figures and Tables

**Table 1:** Clinical and virological parameters of HIV-2 infected patients analyzed in this study.

Subject	Viral load^1^	CDC stage^2^	HIV-2 Group	CA amino acid position^3^		
				119	159	178
HIV-2_ROD_ prototype			A	P	P	P
#13	<99	A	B	G	P	T
#14	<99	A	A	P	S	P
#15	<99	A	B	P	P	A
#18	<99	A	B	A	P	A
#10	862	C	B	V	P	P
#H1	598	C	A	A	P	P
#H4	5398	C	A	Q	S	P
#H5	500	C	A	A	S	P
#H8	2059	C	B	A	P	P

^1^Viral load on sampling date, copies/ml.

^2^According to CDC classification system for HIV infection.

^3^According to the HIV-2 _ROD_ numbering (see Supplemental Fig. 1).
