# Supplementary material for: Cyclophilins and nucleoporins are required for infection mediated by capsids from circulating HIV-2 primary isolates
Source: Sci Rep. 2017 Mar 27;7:45214. doi: 10.1038/srep45214 (PMC5366920; doi:10.1038/srep45214)
Supplement: Supplementary Information [file srep45214-s1.pdf]

SUPPLEMENTARY INFORMATION

Cyclophilins and nucleoporins are required for infection mediated by capsids from circulating HIV-2 primary isolates

João I. Mamede[1 #], Florence Damond[2], Ariel de Bernardo[1], Sophie Matheron[2], Diane Descamps[2], Jean-Luc Battini[1], Marc Sitbon[1] and Valérie Courgnaud[1*]

Supplemental figures legends

Figure S1

Comparison of CA sequences found in patients with the HIV-2_{ROD} prototype. Sequence alignment of matrix and capsid amino acid residues of HIV-2_{ROD} prototype with those of HIV-2 primary isolates found in aviremic patients, in blue (#13, #14, #15 and #18), or viremic patients, in red (#10, #H1, #H4, #H5 and #H8). We cloned and sequenced around 8-10 clones for each patient sample and chose the most frequent sequence. Dots represent fully conserved residues and deletions are indicated with dashes. Numbering of MA and CA residues is indicated following HIV-2 $_{ROD}$ sequence. Also indicated are the beginning of CA and the CypA-binding loop. Proline residues corresponding to HIV-2_{ROD} position 119, 159 and 178 are in bold and marked with a star.

Figure S2

Monitoring of protein expression and infection. (A) Western blot analysis of the different HIV-2 CA chimera proteins. Viral supernatants were harvested 48h after transfection of HEK-293T cells with the wild-type or chimeric SIVmac *gag-pol* expression vectors, a SIVmac-based GFP retroviral vector and the vesicular stomatitis virus G expression vector. Virions were pelleted by ultracentrifugation through a 20% sucrose cushion and CA proteins were detected using serums from HIV-2 infected patients. B) Infection efficiency of serial dilutions

for each HIV-2 chimera in dunni cells is measured as percent of GFP-positive cells, 48h after infection. Absolute titers for all viruses were as follows : (% GFP : SIVmac = 75.2; ROD=65.3; #14=15.23; #15=28.4; #18=28.81; #10=65.2; #H1=80.5; #H4=46.8; H5=78.2; #H8=72). C) Stable expression of TRIM5alpha and TRIM5cyp fusion proteins in dunni cell lines. Cell extracts were obtained from control dunni cells and dunni cells transduced with vectors encoding HA-tagged TRIM5alpha from human (WT or mutant 1/1 and 1/6P479L - hu-TRIM5alpha from 2 independent populations of dunni cells that stably express hu-TRIM5alpha P479L); TRIM5CypA proteins from owl monkey (owl-TRIMCypA), *Macaca mulatta* (mamu-TRIMcypA) and *Macaca fascicularis* (mafa-TRIMcypA). TRIMCypNup358 corresponds to a synthetic TRIMCyp protein obtained by fusing TRIM5 RBCC of TRIMCypA-owl with the human CypA protein. Immunoblotting was performed with monoclonal antibodies directed against HA.

```
HIV.2ROD    MGARNSVLRGKKADELERIRLRPGGKKKYRLKHIVWAANKLDRFGLAESLLESKEGCQKILTVLDPMVPTGSENLKSLFN   MA 80
#13         ........S...T....KV........R.M...VI....E.................R......A.L...........Y.
#14         .................K.....................E....................I.....L.............
#15         ........S...T....KV........E.M...V...V.EM.......T...............A.L.............
#18         ........S...T....KV......R...C....I..V.E.................H......A.L...........L.
#10         ........S...T.Q..KV..........C....I....EM.......T...............A.L.............
#H1         ..RA........L................K.........E..........................L...........Y.
#H4         .................K.........Q...........E.....................S..N.L.............
#H5         .................K.........R...........E........................N.L.............
#H8         ........S...T....KV..........Q...VI..V.EM.......T...............A.LE............
                                                                    CA→
HIV.2ROD    TVCVIWCIHAEEKVKDTEGAKQIVRRHLVAETGTAEKMPSTSRPTAPSSEKGGNYPVQHVGG-NYTHIPLSPRTLNAWVK   CA 24
#13         .......L...T......E..KLAQS..M.D.--EK.T.TADK.A.T..GR--.....QIA.-..V.V............
#14         ......................T.Q...A...K..KE..N....I..P.G....F...Q...-....V............
#15         L......L...V......E..KAAQS..AVD.---..T.A........GGR--.....Q.A.-..V.V............
#18         .....Y.L..........E..K.AQS...VN.---....A.......P.GR--.....QIA.-..V.V............
#10         .....Y.L..........E..K.AQS..A.S.---..ASA.........GR--.....Q.A.-....L............
#H1         ........................Q...A..NK......N.......P.G....F...Q...-..S..............
#H4         ....V.............E..KV.Q......................P.G---.....QTA.G..V.V............
#H5         ....T.............E..KLAQ......A......--........P.GGR..F...QTA.G..V.V............
#H8         L......L...V......E..KAAQS....N.---..T.A.........GG--.....Q.A.-..L.V............
                                                                        CypA binding loop
HIV.2ROD    LVEEKKFGAEVVPGFQALSEGCTPYDINQMLNCVGDHQAAMQIIREIINEEAAEWDVQHPIPGPLPAGQLREPRGSDIAG   CA 104
#13         ..........I........................E.......V.....D...D..Q...S...................
#14         ...D.R.....................................V.........D..A.......................
#15         .I.D...............................E.................D..Q...V..........D........
#18         ...D...............................E.................D..Q...V..........D........
#10         ...................................E.................D..Q..............D........
#H1         ........................................................T.......................
#H4         ...D.................................................D..A..............D........
#H5         ...D.................................................D..A..............D........
#H8         .I.................................E.................D..Q.......................
                          *                                       *                  *
HIV.2ROD    TTSTVEEQIQWMFRPQNPVPVGNIYRRWIQIGLQKCVRMYNPTNILDIKQGPKEPFQSYVDRFYKSLRAEQTDPAVKNWM   CA 184
#13         ........L...Y.G..SI...........L.......V...I....V.........................T..RT..
#14         .....D......Y.........................................S.........................
#15         ............Y..................................V.........................A......
#18         ............Y.A................................V.........................A......
#10         ............Y.VP..............L...............................................K..
#H1         ..............A.................................................................
#H4         ....PD......Y.Q................................V......S.........................
#H5         .....D......Y.A................................V......S.........................
#H8         .....D......Y.A..........................................G......................

HIV.2ROD    TQTLLVQNANPDCKLVLKGLGMNPTLEEMLTACQGVGGP   CA 223
#13         .E.......D............PH...........I...
#14         ...................................I...
#15         .....I.............................I...
#18         .....I.............................I...
#10         .....I.............................I...
#H1         ...................................I...
#H4         ...................................I...
#H5         .....I.............................I...
#H8         .....I.............................I...
```

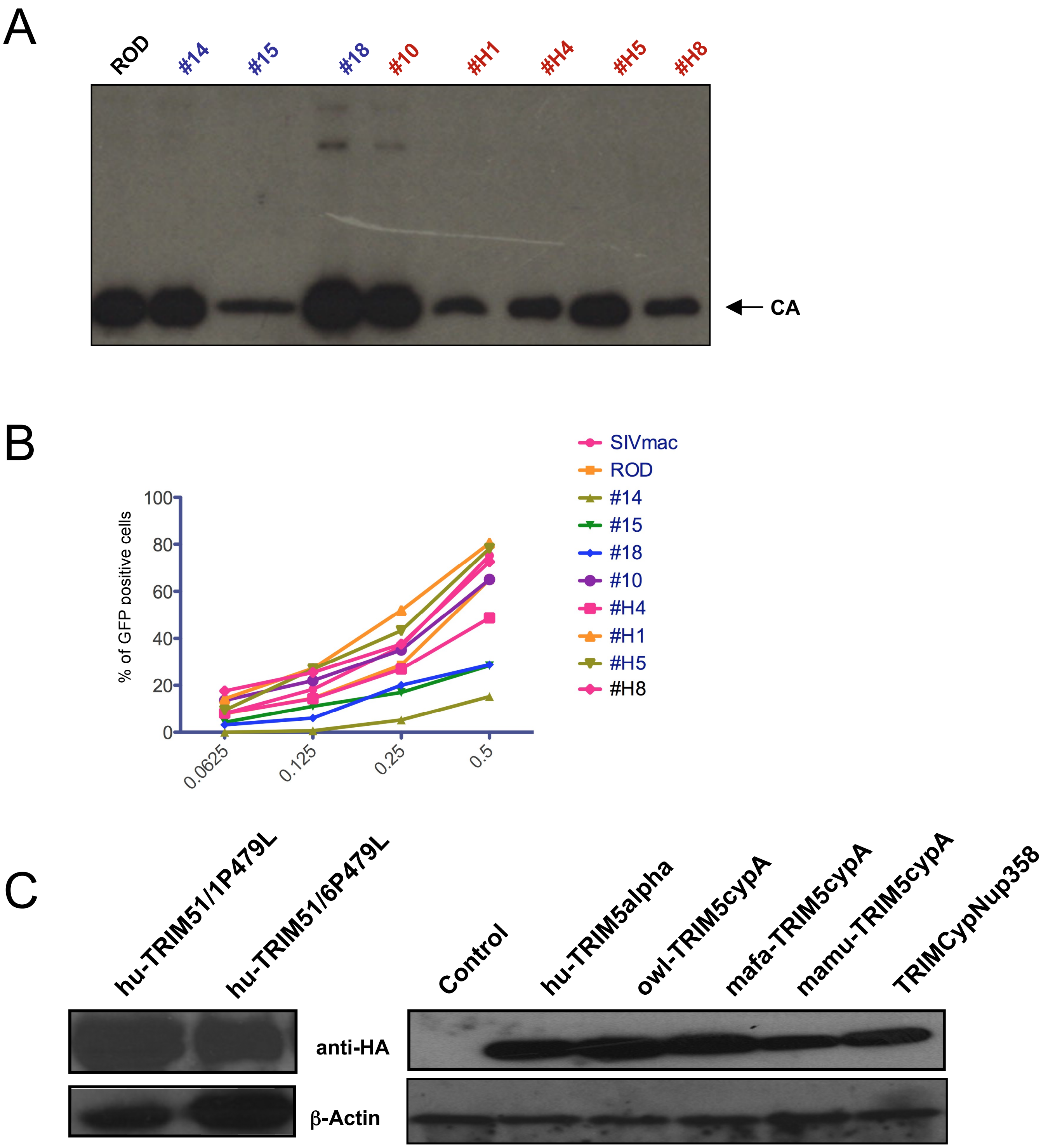
A
ROD
#14
#15
#18
#10
#H1
#H4
#H5
#H8
CA
B
% of GFP positive cells
100
80
60
40
20
0
0.0625
0.125
0.25
0.5
SIVmac
ROD
#14
#15
#18
#10
#H4
#H1
#H5
#H8
C
hu-TRIM51/1P479L
hu-TRIM51/6P479L
Control
hu-TRIM5alpha
owl-TRIM5cypA
mafa-TRIM5cypA
mamu-TRIM5cypA
TRIMCypNup358
anti-HA
β-Actin
